# Novel Phenotype in Unbalanced 7;9 Translocation with Critical Incidental Finding

**DOI:** 10.1155/2022/7510079

**Published:** 2022-05-17

**Authors:** Julie Fischer, Luis Rohena

**Affiliations:** ^1^Department of Pediatrics, Brooke Army Medical Center, Fort Sam Houston, TX, USA; ^2^Division of Medical Genetics, Department of Pediatrics, Brooke Army Medical Center, Fort Sam Houston, TX, USA; ^3^Department of Pediatrics, UT Health San Antonio, Long School of Medicine, San Antonio, TX, USA

## Abstract

This case discusses a now 13-year-old boy who underwent chromosome analysis and fluorescence in situ hybridization (FISH) for subtelomeric rearrangements due to dysmorphic features at birth. This testing revealed a diagnosis of an unbalanced 7;9 translocation resulting in monosomy for 7q34-qter and trisomy for 9pter-p21, which resulted in a very complex medical course. At the age of 12, due to persistent complex neurodevelopmental concerns, the patient was referred by neurology for whole-exome sequencing. This testing revealed an incidental pathogenic heterozygous *KCNH2* deletion, which is associated with long QT-syndrome type II. Prior to this point, the patient had no symptoms of long QT syndrome and had multiple EKGs with normal QT intervals. However, due to this association, the patient underwent Holter monitoring, which revealed clinical evidence of long-QT syndrome type II. Preventative treatment was then initiated and the patient remains asymptomatic. This case expands on the phenotype of this patient's unbalanced 7;9 translocation as well as highlights the importance of secondary findings in genetic testing.

## 1. Introduction

LQTS is a disorder of cardiac repolarization characterized by QT prolongation and T-wave abnormalities on EKG that predisposes the patient to sudden cardiac death [1]. Diagnosis is made by findings of QTc prolongation in the absence of provoking factors (i.e., QT-prolonging drugs and electrolyte derangement) or molecular genetic testing [2].

There are 15 genes that have been associated with LQTS, the most common of which are *KCNQ1* (LQT1), *KCNH2* (LQT2), and *SCN5A* (LQT3) [2]. The majority of cases of LQTS are inherited in an autosomal-dominant manner. The corrected QT interval on resting EKG is not entirely sensitive nor specific for detecting LQTS. Approximately 25% of individuals with LQTS that have genetic testing confirming a pathogenic variant will have a QTc within the normal range, known as concealed LQTS [3]. Cardiac events in LQTS often have genotype-specific triggers [4] Events due to LQTS type 2 are typically triggered by auditory stimuli and emotional stress [4].

There are multiple molecular genetic testing strategies that may be used to identify LQTS. These include a multigene panel, single-gene testing, and more comprehensive genomic testing [2]. 25–30% of LQTS can be attributed to mutations in *KCNH2* [2]. 97–98% of pathogenic variants are detectable by sequence analysis, while only 2–3% are detectable by gene-targeted deletion/duplication analysis (copy number analysis) [2].

## 2. The Patient

The patient was first evaluated by genetics on day of life 1 due to microcephaly and dysmorphic features. The patient was born at 41 weeks gestational age to a 33-year-old G3P3 mother and a 27-year-old father with a pregnancy complicated by a maternal serum triple screen revealing a Down syndrome risk of 1 : 182 (age-related risk 1 : 411). The parents declined to proceed with amniocentesis for definitive testing. Prenatal ultrasounds were reported as normal without suggestive signs of congenital anomalies or Down syndrome, although it is not abnormal for an infant with Down syndrome to have normal prenatal imaging. At birth, dysmorphic facies were noted to be inconsistent with Down syndrome. The patient spent 2 weeks in the NICU for poor feeding prior to discharge home. The parents were in good health. The rest of the family history was unremarkable.

Physical exam at birth was significant for head circumference <3^rd^ percentile, large ears, a prominent nose with a distinct horizontal crease, and slight retrognathia. Fluorescence in situ hybridization (FISH) for subtelomeric rearrangements and a chromosome analysis were performed, and they showed findings remarkable for an unbalanced 7;9 translocation and karyotype 46, XY, der(7)t(7;9)(q34.1; p21) pat. The parents underwent testing which revealed that this was secondary to the father's balanced translocation. The patient's full sister was tested and found to have normal chromosomes. Their families were notified of the patient's chromosomal anomaly, but no other family members have proceeded with genetic testing.

**Figure 1:**
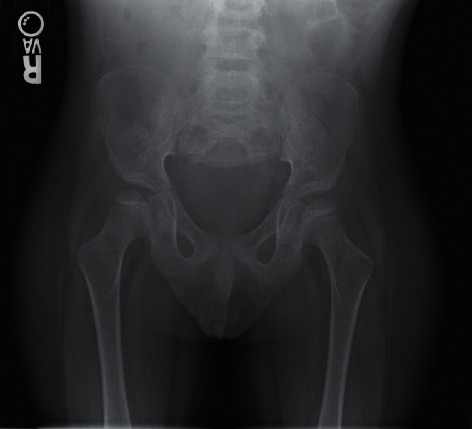
Skeletal survey revealing truncation of the caudal sacrum.

**Figure 2:**
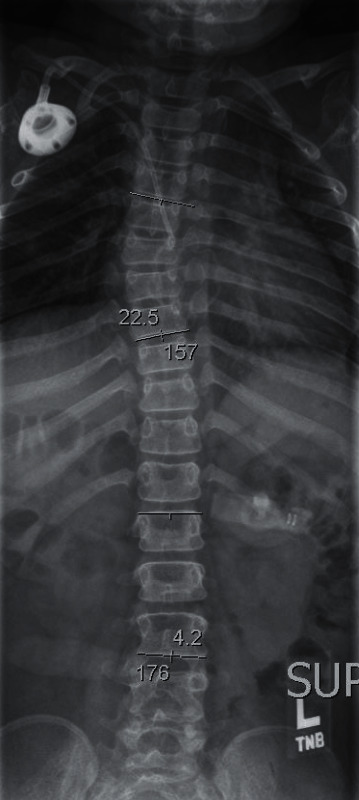
Scoliosis survey revealing 23 degrees of dextrocurvature of the thoracic spine, 4 degrees of levocurvature of the upper lumbar spine, and diffuse osseous demineralization.

**Figure 3:**
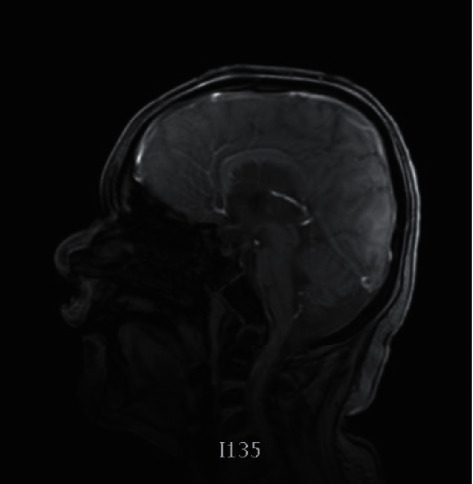
Brain MRI revealing dysgenesis of the corpus callosum, partially empty sella, and third ventricular enlargement.

The patient has experienced multiple medical conditions throughout childhood, to include undescended testes and chordee requiring surgery, malrotation requiring a Ladd's procedure, severe developmental delays, partial sacral agenesis (Figure 1) with a congenital sacral mass requiring resection, hypertrophic scars, cholesteatoma, cataracts, astigmatism, amblyopia, bilateral sensorineural hearing loss requiring hearing aids, obstructive sleep apnea, eustachian tube dysfunction requiring PE tube placement, mineralocorticoid deficiency, growth hormone deficiency, hypotonia, neurogenic bladder, intractable epilepsy requiring ketogenic diet, spastic quadriplegia requiring Botox, syringomyelia, tethered cord, thoracic and lumbar neuromuscular scoliosis (Figure 2), chronic lung disease, constipation, gastroesophageal reflux disease with eosinophilic gastroenteritis, swallowing dysfunction, G-tube dependence with minimal oral feeds, osteopenia and recurrent fractures requiring treatment with bisphosphonates and growth hormone, congenital mitral regurgitation, mitral valve prolapse, and bicuspid aortic valve. He also has multiple abnormalities on brain MRI to include ventriculomegaly, dysgenesis of the corpus callosum, partially empty sella, hypoplasia of the cerebellar vermis, arachnoid cyst, and underdeveloped hippocampi (Figure 3).

At age 10, endocrinology recommended to undergo SNP-Array testing to further characterize the extent of chromosomal involvement, particularly to identify deletions and duplications that may correlate with phenotype. SNP-array testing revealed monosomy for 7q34-qter (144,859,138–159,119,707) and trisomy for 9pter-p21 (203,861–27,009,773). He also received *CYP11B2* testing due to electrolyte disturbances, which was negative.

At age 12, he was referred by neurology for further genetic testing as his neurologist did not believe the complexity of his issues could be fully explained by his chromosomal rearrangement. He underwent whole-exome sequencing, which identified an incidental, likely pathogenic, heterozygous *KCNH2* deletion (7q36.1(150,944,955–150,978,320)x1), which was not detected in either parent. Mutations in this gene are associated with autosomal-dominant long QT syndrome type II.

The patient had previously received electrocardiograms as part of his routine cardiology monitoring, all of which were noted to have QT intervals within the normal range. However, following the identification of the *KCNH2* deletion, the patient was referred for further subspecialty evaluation. At that time, a 12-lead electrocardiogram was notable for a QT interval of 507 ms, with a repeat electrocardiogram revealing a QT interval of 525 ms. A 24-hour Holter monitor was recommended, which confirmed the diagnosis of long QT-syndrome type II. As a result, beta-blockade therapy was initiated, active QT-prolonging medications were discontinued, the family was provided a list of medications that should be avoided in the future, and family members were offered testing.

## 3. Molecular and Cytogenic Methods

The methods used to investigate this patient included microarray, exome sequencing, and MTHFR genotyping. SNP microarray analysis was performed using the Affymetrix Cytoscan HD platform, which uses over 743,000 SNP probes and 1,953,000 NPCN probes with a median spacing of 0.88 kb. 250 ng of total genomic DNA extracted from lymphocytes is digested and amplified using a GeneAmp PCR System 9700. PCR products are purified and quantified using NanoDrop 8000. Purified DNA is fragmented and biotin labeled and hybridized to the Affymetrix Cytoscan HD GeneChip. Data are analyzed using Chromosome Analysis Suite. The analysis is based on the GRCh37/hg19 assembly [5].

Exome sequencing was performed via Invitae Boosted Exome. Per the manufacturer's report, this technology detects single nucleotide variants, indels less than 50 bp, and intragenic copy number variants across >18,000 genes, which are called high quality (depth ≥20x). Joint calling is performed to maximize sensitivity. It is able to detect deletions and duplications spanning 4 exons or more with high confidence. However, smaller events may be detected and reported if sufficient resolution exists. In addition, to ensure high sensitivity and specificity, the exome is sequenced to an average depth of 150x (per base) [6].

## 4. Discussion

The patient's 7q deletion is over 12 Mb in size and contains 149 genes, 72 of which are present in the OMIM catalogue, and of these, 17 are associated with known disease. Patients with deletions similar to those of the patient described have a broad spectrum of largely overlapping abnormalities. The patient's 9p duplication is over 26 Mb in size and contains 229 genes, 90 of which are present in the OMIM catalogue, and of these, 20 are associated with known disease. Similarly, patients with similar duplications have a broad spectrum of largely overlapping abnormalities [7].

Using the Decipher Database, 226 individuals were identified with partial overlap of our patient's 7q deletion and 2 were identified with complete overlap [8]. Supplemental Table 1 displays aggregate data from the Decipher Database of individuals with partial overlap of 7q35 and 7q36.3 deletions (144,859,138–159,199,707), and Supplemental Table 2 displays aggregate data from the Decipher Database of individuals with complete overlap of 7q35 and 7q36.3 deletions (144,859,138–159,199,707), both with phenotypic features that are present in our patient, highlighted in yellow. In addition, 21 individuals were identified with partial overlap of our patient's 9p duplication and 1 was identified with complete overlap. Supplemental Table 3 displays aggregate data from the Decipher Database of individuals with a partial overlap of 9p21.2 duplication (203,861–27,009,773), and Supplemental Table 4 displays aggregate data from the Decipher Database of individuals with a complete overlap of 9p21.2 duplications (203,861–27,009,773), both with phenotypic features that are present in our patient, highlighted in yellow.

The patient presents with several findings common to those previously reported, including seizures, hypotonia, spasticity, mitral regurgitation, microcephaly, flat occiput, deeply set eyes, prominent glabella, intellectual disability, global developmental delay, sensorineural hearing loss, large earlobes/macrotia, prominent ear helix, low-set ears, constipation, gastroesophageal reflux, intestinal malrotation, cryptorchidism, chordee, clinodactyly of the 5^th^ finger, short digits, scoliosis, partial sacral agenesis, and short/bulbous/prominent nose. However, our patient exhibits multiple phenotypic features not previously reported, including hypertrophic scars, cholesteatoma, cataracts, astigmatism, amblyopia, obstructive sleep apnea, eustachian tube dysfunction, neurogenic bladder, syringomyelia, tethered cord, chronic lung disease, eosinophilic gastroenteritis, recurrent fractures, mitral valve prolapse, bicuspid aortic valve, and tented upper vermilion. In addition, there were no reports of long QT syndrome or EKG abnormalities in patients with similar genotypes.

Due to our patient's complex course, he was referred for more comprehensive genetic testing, which included whole-exome sequencing. This testing did not reveal a genetic explanation for his course, which is why we can confidently expand on this phenotype. However, an incidental *KCNH2* deletion was identified, which led ultimately to further work-up and clinical confirmation of the diagnosis of long QT syndrome type II. This allowed for appropriate interventions and treatment prior to the potentially life-threatening clinical manifestations of this syndrome. It also allowed for family members to consider genetic testing. Affected individuals with autosomal-dominant forms most often have a parent who also harbors the pathogenic variant, as the proportion of LQTS secondary to a *de novo* mutation is small [2].

The question of whether to include incidental findings in genomic testing has historically been a controversial one. This is particularly true when the patient is a minor and consent for the testing is obtained by the parent. In 2013, the ACMG recommended the identification and return of incidental findings from a minimum set of 56 actionable genes as a part of clinical genomic testing [9]. In 2014, the ACMG revised their guidelines to allow patients to opt out of receiving this information [10]. These guidelines were further revised in 2016 with the addition of four genes and the removal of one gene, for a total of 59 medically actionable genes recommended for return in clinical genomic sequencing [11]. Fortunately, for our patient, the *KCNH2* deletion is included in the list of these 59 genes to be reported unless the patient specifically ops out [11].

However, these 59 genes include only those determined by the ACMG to be the most medically actionable. There are many more genes with pathologic implications that are not included in this recommended list. For example, a case report published in 2011 describes a child with refractory inflammatory bowel disease (IBD) who underwent whole-exome sequencing which identified a mutation in the *XIAP* gene, a known cause of the x-linked proliferative disorder. This immunodeficiency is now known to be implemented in a previously unrecognized form of IBD [12]. Had this test been limited to single-gene sequencing, this mutation would not have been identified as it was not on the list of candidate genes at the time [12]. This gene is also not included within the 59 genes recommended for return in the updated ACMG recommendations [13]. Similarly, Amendola et al. analyzed exomes from 6503 participants from the National Heart, Lung, and Blood Institute Exome Sequencing Project for variants in 112 medically actional genes and found that 2.0% of adults of European ancestry and 1.1% of adults of African ancestry could be expected to have actionable highly penetrant pathogenic or likely pathogenic single-nucleotide variants [13]. However, when they only analyzed those pathogenic variants included in the ACMG recommendations for reporting of incidental findings (56 genes at the time of analysis), the proportion of individuals with returned incidental findings would only be 0.7% in those of European ancestry and 0.5% in those of African ancestry [13].

Controversy exists as to whether the risks of disclosing further incidental findings, particularly nonmedically actionable findings, outweigh the benefits of discovery. Because of this, many advocate for the power of patient choice. However, do patients truly want to know? Christenhusz et al. gathered data from eight diverse focus groups discussing the genomic sequencing of children and found that, even when the information gained could be ambiguous, fail to lead to any treatment, and be potentially harmful, the majority of patients reported that they would prefer to know about these findings [14]. However, Roche and Berg describe that, in their preliminary experience with North Carolina Clinical Genomic Evaluation by NextGen Exome Sequencing, participants who had been randomized to make a decision regarding nonmedically actionable secondary findings, only a minority actually requested them [15]. This suggests that even when participants express an intention to be made aware of such secondary findings, this may not reflect an unequivocal desire to learn them [15].

Therefore, we advocate for importance of continued genetic investigation even in patients with identified genetic abnormalities, particularly as testing continues to advance. In addition, we provide support for the importance of revealing incidental findings in genomic testing, as well as suggest that disclosure of this information should be guided by patient choice.

## Supplementary Materials

Supplementary Materials Supplemental Table 1: reported clinical features of individuals with partial overlap of 7q35 and 7q36.3 deletions. Supplemental Table 2: reported clinical features of individuals with complete overlap of 7q35 and 7q36.3 deletions. Supplemental Table 3: reported clinical features of individuals with partial overlap of 9p21.2 duplication. Supplemental Table 4: reported clinical features of individuals with complete overlap of 9p21.2 duplication.
