# Supplementary material for: Novel Phenotype in Unbalanced 7;9 Translocation with Critical Incidental Finding
Source: Case Rep Genet. 2022 May 17;2022:7510079. doi: 10.1155/2022/7510079 (PMC9130017; doi:10.1155/2022/7510079)
Supplement: Supplementary Materials — Supplemental Table 1: reported clinical features of individuals with partial overlap of 7q35 and 7q36.3 deletions. Supplemental Table 2: reported clinical features of individuals with complete overlap of 7q35 and 7q36.3 deletions. Supplemental Table 3: reported clinical features of individuals with partial overlap of 9p21.2 duplication. Supplemental Table 4: reported clinical features of individuals with complete overlap of 9p21.2 duplication. [file 7510079.f1.docx]

| Supplemental Table 1: Reported Clinical Features of Individuals with Partial Overlap of 7q35 and 7q36.3 Deletions | | |  |
| --- | --- | --- | --- |
| Behavioral | Autism/ Aggressive Behavior/ Behavior abnormality/ Self-Mutilation | 29 |  |
|  | Attention deficit hyperactivity disorder/ Hyperactivity/ Short attention span | 4 |  |
| Brain/CNS | Seizure/ EEG abnormality | 25 |  |
|  | Ependymoma/ Neoplasm of the central nervous system | 2 |  |
|  | Hypotonia | 18 |  |
|  | Hypertonia | 6 |  |
|  | Aplasia/hypoplasia/dysplasia of the corpus callosum/ Hydrocephalus/ Aplasia/hypoplasia of the cerebellum/ Arnold-Chiari type 1 malformation/ Holoprosencephaly/ Brainstem dysplasia/ Cerebral atrophy/ J-shaped sella turcica/ Cavernous hemangioma/ Cerebral arteriovenous malformation/ Dandy-Walker malformation/ Abnormality of the septum pellucidum/ Anterior pituitary agenesis/ Lissencephaly | 77 |  |
|  | Spastic diplegia/ spasticity | 4 |  |
|  | Bimanual synkinesia/ Abnormality of extrapyramidal motor function/ Meningitis | 3 |  |
| Cardiac | Atrial septal defect/ Patent Ductus Arteriosis/ Aortic regurgitation/ Trucuspid regurgitation/ Ventricular septal defect/ Pulmonary valve atresia/ Pulmonic stenosis/ Pulmonary insufficiency/ Coarctation of the aorta/ Atrioventricular canal defect/ Aortic valve stenosis/ Pulmonary artery hypoplasia/ Tricuspid stenosis | 42 |  |
|  | Tetrology of Fallot/ Hypoplastic left heart/ Single ventricle/ Dextrocardia | 5 |  |
|  | Abnormality of cardiovascular system/ Abnormal ventriculoarterial connection/ Cardiomyopathy | 8 |  |
|  | Mitral regurgitation | 1 |  |
| Craniofacial | Brachycephaly/ Macrocephaly/ Craniosynostosis/ Trigonocephaly/ Thin calvarium/ Extra fontanelles/ Delayed closure of the anterior fontanelle/ Premature closure of fontanelles/ Frontal bossing/ Rigid cranial sutures/ Wide cranial sutures/ Plagiocephaly | 36 |  |
|  | Microcephaly | 71 |  |
|  | Flat occiput | 2 |  |
|  | Upslanted palpebral fissure/ Downslanted palpebral fissures/ Unilateral narrow palpebral fissure/ Short palpebral fissures/ Palpebral edema/ Almond-shaped palpebral fissure | 41 |  |
|  | Microphthalmia/ Epicanthus/ Hypertelorism/ Hypotelorism/ Telecanthus/ Ptosis/ Unilateral ptosis/ | 70 |  |
|  | Deeply set eye | 7 |  |
|  | Thick lower lip vermilion/ Thick upper lip vermilion/ Thin upper lip vermilion/ Thin lower lip vermilion/ Everted lower lip vermilion/ Abnormality of the mouth/ Abnormal oral frenulum morphology/ Downturned corners of mouth/ Wide mouth/ Narrow mouth | 50 |  |
|  | Exaggerated cupid’s bow/ Hypomimic face/ Abnormality of the face/ Abnormal face shape/ Midface retrusion/ Hyperplasia of midface/ Malar flattening/ Malar prominence/ Triangular face/ Pointed chin/ Narrow forehead/ Sloping forehead/ Broad forehead/ Round face/ Small face/ Long face/ Full cheeks/ Facial asymmetry/ Coarse facial features/ Prominent supraorbital ridges | 44 |  |
|  | Short philtrum/ hypoplastic philtrum/ Long philtrum/ Broad philtrum | 16 |  |
|  | Median cleft lip/ Non-midline cleft lip/ Cleft upper lip/ Cleft palate/ Oral cleft/ High palate/ Narrow palate/ Abnormal palate morphology/ Submucous cleft hard plate | 58 |  |
|  | Prominent glabella | 3 |  |
|  | Migrognathia/ Cleft of alveolar ridge of maxilla/ Hypoplasia of the maxilla/ Aplasia of the premaxilla/ Mandibular prognathia | 39 |  |
|  | Macroglossia/ Protruding tongue/ Aplasia/hypoplasia of the tongue/ Prominent palatine ridges/ Aplasia/hypoplasia of the uvula | 6 |  |
|  | Microdontia/ Oligodontia/ Single median maxillary incisor/ Misalignment of teeth/ Gingival overgrowth/ Abnormality of the dentition/ Abnormality of dental morphology | 21 |  |
| Development/Growth | Intellectual disability/ abnormality of higher mental function/ cognitive impairment/ Neurodevelopmental delay/ Learning disability/ Delayed speech and language development/ Absent speech/ Motor delay/ Delayed gross motor development/ Psychomotor retardation | 105 |  |
|  | Global developmental delay | 25 |  |
|  | Short Stature | 64 |  |
|  | Disproportionate short-limb short stature/ upper limb undergrowth/ aplasia/hypoplasia of the extremities/severe limb shortening/ Abnormality of the upper limb/ Abnormality of the lower limb | 6 |  |
|  | Postnatal growth retardation/ Small for gestational age/ Growth delay | 42 |  |
|  | Apraxia/ Dyscalculia/ Impaired visuospatial constructive cognition | 3 |  |
| Ear/ Auditory | Sensorineural hearing impairment/ loss | 4 |  |
|  | Conductive hearing impairment/ Hearing impairment/ hearing abnormality | 5 |  |
|  | Small earlobe/ Anterior creases of earlobe/ Stenosis of the external auditory canal/ Abnormality of the antihelix/ Prominent antihelix/ Abnormality of the outer ear/ Microtia/ Protruding ears/ Abnormality of the pinna/ Aplasia/hypoplasia of the earlobes/ Posteriorly rotated ears/ Abnormality of the outer ear | 53 |  |
|  | Large earlobe | 2 |  |
|  | Prominent ear helix | 1 |  |
|  | Macrotia | 17 |  |
|  | Low-set ears | 29 |  |
| Endocrine | Diabetes mellitus/ Hypoglycemia/ Primary amenorrhea/ Hypothyroidism/ Obesity/ Truncal obesity/ Precocious puberty in males/ Anterior hypopituitarism | 14 |  |
| Gastrointestinal | Constipation | 5 |  |
|  | Aganglionic megacolon/ Intestinal duplication/ Hiatal hernia/ Anal stenosis/ Anal atresia/ Ectopic anus/ Duodenal atresia/ Abnormality of the duodenum/ Omphalocele/ Abnormality of mesentery morphology/ Abnormality of the gallbladder/ Biliary atresia/ Annular pancreas/ Meckel diverticulum/ Abnormal esophagus morphology/ Polysplenia | 29 |  |
|  | Gastroesophageal reflux | 4 |  |
|  | Bowel incontinence/ Protuberant abdomen | 2 |  |
|  | Intestinal malrotation | 2 |  |
| Genitourinary/ Renal | Hypospadias/ Displacement of the urethral meatus/ Abnormality of the urethra/ Urethral stenosis/ Urethral diverticulum/ Micropenis | 35 |  |
|  | Scrotal hypoplasia/ Hydrocele testis/ Abnormality of the labia/ Clitoral hypertrophy/ Abnormality of the genital system | 11 |  |
|  | Cryptorchidism | 12 |  |
|  | Ureteral atresia/ Abnormality of the ureter/ Nephrolithiasis/ Hydronephrosis/ Recurrent urinary tract infections/ Duplicated collecting system/ Fetal pyelectasis Patent urachus/ Urinary incontinence/ Enuresis/ Unilateral renal agenesis/ Renal agenesis/ Renal dysplasia/ Renal hypoplasia/ Abnormality of the kidney/ Enlarged kidney/ Horseshoe kidney/ Multiple renal cysts/ Renal cyst/ Vesicoureteral reflux/ Hypertrophy of the urinary bladder/ Abnormality of the bladder/ Polyuria | 52 |  |
|  | Chordee | 1 |  |
| Hair | Frontal upsweep of hair/ Abnormality of scalp hair/ Low anterior hairline/ Low posterior hairline/ High anterior hairline/ Sparse hair/ Hypertrichosis/ Generalized hirsutism | 26 |  |
|  | Thick Eyebrow/ Highly arched eyebrow/ Medial flaring of the eyebrow/ Synophrys | 8 |  |
| Hands/ Feet | Short palm/ Broad palm/ Large hands/ Small hands/ Abnormal dermatoglyphics/ Single transverse palmar crease/ Deep palmar crease/ Small hypothenar eminence/ Small thenar eminence/ Ulnar deviation of the hand/ Abnormality of the hand/ Edema of the dorsum of hands | 28 |  |
|  | Hyperextensibility of the finger joints/ Camptodactyly of finger/ Overlapping fingers/ Prominent fingertip pads/ Abnormal thumb morphology/ Proximal placement of thumb/ Broad thumb/ Short metacarpal/ Preaxial hand polydactyly/ Polydactyly/ Arachnodactyly/ Tapered finger/ Ulnar deviation of finger/ Brachydactyly | 35 |  |
|  | Clinodactyly of the 5^th^ finger | 1 |  |
|  | Finger clinodactyly | 11 |  |
|  | Short digit | 2 |  |
|  | Overlapping toe/ Long toe/ Abnormality of toe/ Hammertoe/ Preaxial foot polydactyly/ Foot polydactyly/ Long foot/ Short foot/ Sandal gap/ 2-3 toe syndactyly/ Toe syndactyly/ Deep plantar creases/ Edema of the dorsum of feet/ Broad foot | 38 |  |
|  | Hyperconvex nail/ Nail dysplasia/ Anonychia | 5 |  |
| Musculoskeletal | Scoliosis/ thoracolumbar scoliosis | 11 |  |
|  | Congenital kyphoscoliosis/ kyphosis/ Abnormality of the lumbar spine/ Vertebral fusion/ Hemivertebrae/ Abnormal vertebral morphology/abnormality of the vertebral column/ Vertebral segmentation defect/ Unossified vertebral bodies | 13 |  |
|  | Absence of sacrum/ Abnormal ossification of the pubic bones/ Hypoplastic ilia/ Hip dysplasia/ Hip dislocation/ Hip contracture/ Abnormality of lower limb joint/ Genu valgum/ Pes planus/ Knee flexion contracture/ Rocker bottom feet/ Metatarsus valgus/ Metatarsus adductus/ Talipes equinovarus/ Abnormal tibia morphology/ Hyperextensibility of the knee/ Wide pubic symphysis | 60 |  |
|  | Aplasia/hypoplasia of the sacrum/ hemisacrum/ sacral segmentation defect/ abnormal sacrum morphology | 16 |  |
|  | Elbow Dislocation/ Cubitus valgus | 4 |  |
|  | Multiple exostoses/ Delayed skeletal maturation/ Flexion contracture | 12 |  |
|  | Narrow chest/ Barrel-shaped chest/ Pectus excavatum/ Pectus carinatum/ Abnormality of the ribs/ Missing ribs/ Thin ribs/ Short sternum/ Abnormality of the thorax/ Long thorax/ Short thorax | 21 |  |
| Nasal | Aplasia of the nose/ Depressed nasal bridge/ Depressed nasal ridge/ Convex nasal ridge/ Wide nasal bridge/ Prominent nasal bridge/ Abnormality of the nasal bridge/ Broad nasal tip/ Abnormality of the nasl tip/ Anteverted nares/ Choanal atresia/ Choanal stenosis/ Midnasal stenosis/ Absent nasal septal cartilage/ Abnormality of the nasal septum/ Single naris/ Narrow naris/ Proboscis/ Underdeveloped nasal alae/ Abnormality of the nose/ Aplasia of the nose/ Abnormality of the columella | 73 |  |
|  | Prominent nose | 5 |  |
|  | Bulbous nose | 17 |  |
|  | Short nose | 19 |  |
| Ocular | Strabismus/ Alternating esotropia/ Esotropia/ Nystagmus | 18 |  |
|  | Blue Sclera/ Coloboma/ Iris coloboma/ Optic nerve coloboma/ Optic disc hypoplasia/ Abnormality of the eye/ Rod-cone dystrophy/ Abnormal pupil morphology/ Anisocoria/ Ectopia lentis/ Microcornea/ Persistent pupillary membrane/ Sclerocornea/ Long eyelashes | 30 |  |
|  | Cyclopia/ Proptosis/ Anophthalmia | 13 |  |
|  | Visual impairment/ Hypermetropia/ Blindness | 4 |  |
| Other | Presacral teratoma/ Spinal cord tumor/ Neuroblastoma/ Multiple lipomas | 9 |  |
|  | Oligohydramnios/ Polyhydramnios/ Premature birth/ Abnormality of prenatal development or birth/ Breech presentation/ Caesarian section/ Intrauterine growth retardation/ Preeclampsia | 51 |  |
|  | Inguinal hernia/ Umbilical hernia/ Abnormal umbilicus morphology/ Congenital diaphragmatic hernia | 17 |  |
|  | Systemic lupus erythematosus/ Sleep disturbance/ Hypertension/ Adrenal hypoplasia/ Situs inversus totalis/ Tremor/ Recurrent infections/ Abnormal lung lobation/ Respiratory distress/ Abnormality of the upper respiratory tract/ Spina bifida/ Spina bifida occulta/ Ataxia | 24 |  |
|  | Nasogastric tube feeding in infancy/ Feeding difficulties in infancy/ Failure to thrive/ Impaired mastication | 15 |  |
|  | Wide intermammillary distance/ Supernumerary nipple/ Hypoplastic nipples/ Sacral dimple/ Diastasis recti/ Cystic hygroma | 20 |  |
|  | Short neck/ Redundant neck skin/ Webbed neck/ Broad neck | 40 |  |
| Skin | Skin Abnormality/ Hemangioma/ Facial hemangioma/ Eczema/ Cutis marmorata/ Neoplasm of skin/ Nevus/ Spotty hyperpigmentation/ Hypopigmentation of the skin | 16 |  |

Aggregate data from Decipher Database of individuals with partial overlap of 7q35 and 7q36.3 deletions (144,859,138-159,199,707)

Phenotypic features present in our patient highlighted in yellow

|  | Supplemental Table 2: Reported Clinical Features of Individuals with Complete Overlap of 7q35 and 7q36.3 Deletions | |  |
| --- | --- | --- | --- |
| Brain/CNS | | Holoprosencephaly/ Hypoplasia of the corpus callosum | 2 |
|  | | Microcephaly | 1 |
| Craniofacial | | High palate | 1 |
|  | | Hypertelorism | 1 |
| Development/Growth | | Delayed speech and language development | 1 |
|  | | Intellectual disability | 2 |
|  | | Short stature | 1 |
|  | | Scoliosis | 1 |
| Musculoskeletal | | Vertebral segmentation defect | 1 |
| Ocular | | Strabismus/ Optic atrophy | 2 |
|  | | Optic atrophy | 1 |

Aggregate data from Decipher Database of individuals with complete overlap of 7q35 and 7q36.3 deletions (144,859,138-159,199,707)

Phenotypic features present in our patient highlighted in yellow

|  | Supplemental Table 3: Reported Clinical Features of Individuals with Partial Overlap of 9p21.2 Duplication | |  |
| --- | --- | --- | --- |
| Brain/CNS | | Hydrocephalus/ Ventriculomegaly/ Abnormality of the choroid plexus/ Diffuse cerebral calcification/ Dilated thirtd ventricle/ Mild fetal ventriculomegaly/ Agenesis/hypoplasia of the corpus callosum | 9 |
|  | | Hypotonia | 3 |
| Cardiac | | Ventricular septal defect/ Patent ductus arteriosis/ Tricuspid regurgitation/ Atrial septal defect/ Abnormal heart morphology | 6 |
| Craniofacial | | Frontal bossing/ Macrocephaly/ Trigonocephaly/ Prominent metopic ridge/ Plagiocephaly | 5 |
|  | | Microcephaly | 2 |
|  | | Migrognathia/ Microretrognathia/ Pointed chin | 4 |
|  | | Downslanted palpebral fissures/ Upslanted palpebral fissures/ Short palpebral fissures/ Periorbital fullness/ Hypertelorism | 6 |
|  | | Downturned corners of mouth/ Thin upper lip vermilion/ Hypoplastic philtrum/ Smooth philtrum | 6 |
|  | | Coarse facial features/ Abnormal face shape | 2 |
| Dental | | Widely spaced teeth | 1 |
| Development/ Growth | | Tall stature | 1 |
|  | | Short stature | 1 |
|  | | Intellectual disability/ abnormality of higher mental function/ Motor delay | 9 |
|  | | Global developmental delay | 3 |
| Ear/ Auditory | | Low-set ears | 1 |
|  | | Protruding ear/ Microtia | 2 |
|  | | Macrotia | 1 |
|  | | Hearing impairment | 1 |
| Endocrine | | Hypoglycemia | 1 |
| Gastrointestinal | | Duodenal atresia/ Omphalocele/ Hepatic calcification | 3 |
| Genitourinary/ Renal | | Abnormality of the genital system/ Urethrocele | 2 |
|  | | Renal cyst | 1 |
| Hair | | High anterior hairline/ Low posterior hairline | 2 |
| Hands/ Feet | | Abnormality of the palmar creases/ Single transverse palmar crease/ Large hands | 4 |
|  | | Clinodactyly of the 5^th^ finger | 1 |
|  | | Aplasia/hypoplasia involving the metacarpal bones | 1 |
|  | | Aplasia/hypoplasia of the toe/ Long foot/ Talipes | 3 |
|  | | Short phalanx of finger | 1 |
| Musculoskeletal | | Accelerated skeletal maturation/ Joint laxity | 2 |
|  | | Scoliosis | 2 |
|  | | Aplasia/hypoplasia of the fibula | 1 |
|  | | Aplasia/hypoplasia of the tibia | 1 |
| Nasal | | Depressed nasal bridge/ Wide nasal bridge/ Anteverted nares | 4 |
|  | | Bulbous nose | 1 |
|  | | Deeply set eye | 1 |
| Ocular | | Nystagmus/ Optic nerve hypoplasia/ Epicanthus | 3 |
| Other | | Cesarian section/ Single umbilical artery/ Intrauterine growth retardation | 6 |
|  | | Abnormality of ductus venosus blood flow/ Congenital portosystemic venous shunt/ Lymphangioma/ Pleural effusion/ Short neck/ Unsteady gait/ Nasal speech | 8 |
| Skin | | Striae distensae/ Hypomelanotic macule | 2 |

Aggregate data from Decipher Database of individuals with partial overlap of 9p21.2 duplication (203,861-27,009,773)

Phenotypic features present in our patient highlighted in yellow

|  | Supplemental Table 4: Reported Clinical Features of Individuals with Complete Overlap of 9p21.2 Duplication | |  |
| --- | --- | --- | --- |
| Brain/CNS | | Hypotonia | 1 |
| Craniofacial | | Micrognathia | 1 |
| Development/Growth | | Global developmental delay | 1 |
| Hand/Feet | | Adducted thumb | 1 |
| Musculoskeletal | | Scoliosis | 1 |
| Other | | Intrauterine growth retardation | 1 |
| *Aggregate data from Decipher Database of individuals with complete overlap of 9p21.2 duplication (203,861-27,009,773)*  Phenotypic features present in our patient highlighted in yellow | | | |
